# A Meta-Analysis on Outcomes of Zanidatamab Plus Chemotherapy in Patients with Advanced HER2-positive Gastroesophageal Adenocarcinoma

**DOI:** 10.1007/s12029-026-01608-6

**Published:** 2026-10-15

**Authors:** Henrique Jin Son Kim, Murilo Lauria Freire Ribeiro, Leonardo Gil-Santana, Guilherme Rossato de Almeida, Angelo Borsarelli Carvalho Brito, Tiago Cordeiro Felismino

**Affiliations:** 1 Division of Oncology, Department of Medical Oncology, A. C. Camargo Cancer Center, 211 Professor Antônio Prudente, Liberdade, São Paulo, SP Brazil; 2 Department of Medicine, University of Toronto, Princess Margaret Cancer Centre, University Health Network, Toronto, Canada

**Keywords:** Zanidatamab, Stomach neoplasms, Esophageal neoplasms, Gastroesophageal junction, HER2-positive

## Abstract

**Background:**

Zanidatamab is a novel HER2-targeted antibody that binds to two non-overlapping extracellular domains of the HER2 receptor. Early-phase trials have shown clinical benefit of first-line Zanidatamab in patients with advanced HER2-positive esophageal adenocarcinoma (GEA). We performed a meta-analysis to evaluate the efficacy and safety of zanidatamab in this patient population.

**Methods:**

We searched PubMed, Embase, Cochrane, ASCO, and ESMO databases for studies examining zanidatamab as first-line treatment in patients with HER2-positive advanced GEA. Outcomes of interest included progression-free survival (PFS); objective response rate (ORR); disease control rate (DCR); duration of response (DOR); and incidence of adverse events (AEs). Analyses were presented as median or proportions in percentages with 95% confidence intervals (CIs). Random-effect models were used for analysis, which were carried out using R software (v4.5.2).

**Results:**

Three clinical trials involving 685 patients with advanced HER2-positive GEA treated with zanidatamab in combination with chemotherapy and/or tislelizumab were included. Overall, the reconstructed pooled median OS was 26.05 months (95% CI: 23.60-30.04), while the pooled median PFS was 12.61 months (95% CI: 11.05–14.60). The pooled ORR was 70% (95% CI, 67–74), DCR was 96% (95% CI, 78–100), and median DOR was 17.11 months (95% CI, 13.07–22.40). Treatment-related diarrhea leading to dose reduction was observed in 16% (95% CI, 1%-75%) of patients. Treatment discontinuation due to any cause rate was 20% (95% CI, 3%-44%). Serious treatment-emergent AEs occurred in 66% (95% CI, 50%-81%) of patients. Common any-grade AEs included diarrhea (89%; 95% CI, 75%-98%), nausea (60%; 95% CI, 45%-74%), and infusion-related reactions (30%; 95% CI, 19%-43%). Grade ≥ 3 diarrhea, nausea, and treatment-related AEs rates were as follows: 26% (95% CI, 19%-34%), 5% (95% CI, 2%-13%), and 65% (95% CI, 58%-71%), respectively. Other important AEs include immune-mediated AEs (25% incidence rate), left ventricular dysfunction (6% incidence rate), and non-infectious pulmonary toxicity (4% incidence rate).

**Conclusions:**

Our pooled analyses suggest that zanidatamab exhibits meaningful activity as first-line treatment of advanced HER2-positive GEA. Yet, concerning toxicity rates were also consistently reported across studies. Future randomized studies are needed to further explore its activity and safety profile in this population.

**Supplementary Information:**

The online version contains supplementary material available at https://doi.org/10.1007/s12029-026-01608-6.

## Introduction

Gastro-esophageal adenocarcinoma (GEA) constitutes a group of malignancies that includes adenocarcinomas of the stomach, esophagus, and the gastro-esophageal junction (GEJ) [1]. It ranks as the fifth most commonly diagnosed cancer and is also the fifth leading cause of cancer-related mortality worldwide [2]. Patients with gastro-esophageal adenocarcinoma are often diagnosed at an advanced stage, which is associated with a poor prognosis. Despite recent advancements in systemic therapy, survival outcomes for unresectable or metastatic GEA remain limited. This highlights the need for more effective strategies in biologically selected populations, such as those with HER2-positive disease, which accounts for approximately 20% of gastric cancer patients and 30% of GEJ cancer patients [3, 4]. 

HER2 overexpression or amplification is a clinically relevant biomarker in GEA, since it has been determinant for first-line treatment in this subgroup. The ToGA trial established trastuzumab in combination with platinum and fluoropyrimidine as a standard first-line option for patients with HER2-positive advanced gastric or GEJ cancer [3]. Several studies have evaluated the incorporation of new HER2-targeted treatments, such as pertuzumab, into standard chemotherapy regimens with or without trastuzumab, but these did not demonstrate an increase in overall survival, highlighting the persistent need for more effective HER2-targeted treatments for HER2-positive gastric cancer/GEJC [5]. Recently, combining pembrolizumab with trastuzumab and chemotherapy has led to further improvements in patient outcomes. This combination has been included in current regulatory approvals for individuals with PD-L1-positive tumors, emphasizing the role of HER2-targeted therapy as the foundation of contemporary first-line treatment [6]. However, not all patients derive enduring benefits, and both primary and acquired resistance remain substantial clinical challenges.

Zanidatamab is a novel, humanized bispecific antibody that targets HER2, resembling immunoglobulin G isotype 1. It specifically binds to the juxtamembrane extracellular domain and dimerization domain of HER2. The unique properties of zanidatamab allow it to attach to HER2 at various expression levels, promote receptor cluster formation, and facilitate receptor internalization, ultimately leading to HER2 downregulation. This antibody inhibits tumor cell proliferation, regardless of whether it is dependent on or independent of growth factors, and initiates immune-mediated responses, including antibody-dependent cellular cytotoxicity, antibody-dependent cellular phagocytosis, and complement-dependent cytotoxicity [7]. 

Nonetheless, the current evidence regarding zanidatamab for advanced HER2-positive GEA is scattered across early-phase single-arm trials and emerging phase 3 data, making it challenging to determine the extent and consistency of its benefits and side effects across different studies. Furthermore, survival outcomes, such as OS and PFS, which are more clinically significant than response rates, have not been thoroughly compiled in a pooled analysis. Consequently, we conducted a systematic review and meta-analysis of studies assessing zanidatamab-based first-line treatment in advanced HER2-positive GEA to more accurately define its efficacy and safety profile, with a particular focus on survival outcomes and treatment-related toxicity.

Current evidence on zanidatamab for advanced HER2-positive GEA is spread across early trials and new phase 3 data making it difficult to define the magnitude and consistency of benefit and toxicity across studies. Therefore, we performed a systematic review and meta-analysis to evaluate the efficacy and safety of zanidatamab as a first-line treatment for advanced HER2-positive GEA, with particular emphasis on survival outcomes and treatment-related toxicity.

## Materials and Methods

This systematic review and meta-analysis followed the recommendations of the Cochrane Handbook for Systematic Reviews of Interventions and the Preferred Reporting Items for Systematic Reviews and Meta-Analysis (PRISMA) statement guidelines. We registered the study on PROSPERO under protocol number CRD42026130377.

### Search Strategy and Data Collection

On January 15, 2026, a systematic search was performed on PubMed, Cochrane, Embase, the European Society for Medical Oncology (ESMO), and the American Society of Clinical Oncology (ASCO) databases and was last updated in June 3, 2026. The search terms included “zanidatamab”, “Ziihera”, “ZW25” and “ZW-25”. The entire search is available in Table S2. Relevant reviews and unpublished clinical trials were evaluated for any additional studies. We additionally searched for real-world or observational studies evaluating Zanidatamab in this setting; none were identified.

Two authors (HK and MR) independently screened reports by title and abstract, selected studies for a comprehensive review, collected data from individual studies, and performed the quality assessment. Disagreements were resolved by consensus or by consulting a third author (LS).

### Eligibility Criteria

We included clinical trials (phase II or III) evaluating the efficacy and safety of zanidatamab plus chemotherapy in patients with advanced HER2-positive gastroesophageal adenocarcinoma in first-line setting. There were no restrictions regarding age, gender, histological subtype and number of patients. Abstracts and presentations from conferences were also considered for inclusion. We excluded reviews, case reports, case series, case-control, and preclinical studies. Ongoing studies with results unavailable at the time of the search were excluded.

### Intervention

The intervention of interest was the administration of zanidatamab plus chemotherapy in first-line setting. The dosing regimen varied between 30 mg/kg or 1800 mg in patients weighing < 70 kg and 2400 mg in those weighing ≥ 70 kg, all in an every-three-week schedule. Studies investigating combination therapies were included if they reported relevant efficacy or safety outcomes. A sensitivity leave-one-out analysis was performed to explore the influence of combined therapy studies on the overall effect of our primary outcome of interest.

### Outcomes

The primary outcome of interest was the overall survival (OS). Secondary outcomes included the progression-free survival (PFS), objective response rate (ORR), disease control rate (DCR), duration of response (DOR), and incidence of adverse events (AEs) of any grade, as well as serious adverse events (grade ≥ 3).

### Quality Assessment

The Risk of Bias in Non-randomized Studies of Interventions (ROBINS-I) tool was used to assess the quality of nonrandomized studies [8]. For the randomized controlled trial (RCT), we used the Cochrane Risk of Bias 2 Tool (RoB 2) [9]. Two independent reviewers (HK and GA) carried out this assessment, and disagreements were resolved by consensus between the authors after reviewing the full article. Following this protocol, each study was classified as low, moderate, serious, critical risk of bias or no information based on seven domains: bias due to confounding, selection of participants into the study, classification of interventions, deviations from intended intervention, missing data, in the measurement of outcomes and the selection of the reported result.

### Statistical Analysis

Statistical analysis was performed using R software (version 4.5.2). We used the R packages ´meta´, ´metafor´, and ´weight´. Inverse variance and random-effect models were used for all proportional meta-analyses. We used the number of events per population to carry out proportional meta-analyses, and they were reported in percentages with 95% confidence intervals (CI). Logit transformation was used when the individual study proportion was higher than 0.8 or lower than 0.2. Double arcsine transformation was used in studies where the proportion was zero or one. Heterogeneity was assessed using the I² test.

For survival outcomes (OS and PFS), two authors (HK and MR) independently collected data from published Kaplan-Meier curves using the WebPlotDigitizer web software to extract raw coordinates of time and survival probabilities. We then reconstructed studies’ individual patient data (IPD) through ‘IPDfromKM’ package. Numbers at risk reported in the original publications were incorporated into the reconstruction algorithm. We evaluated the accuracy of reconstructed curves by visual inspection and by quantifying the agreement between reconstructed and digitized survival probabilities using root-mean-square error, mean absolute error and maximum absolute error. Finally, all reconstructed IPDs were merged to create a new dataset. The merged dataset was used to create a new Kaplan-Meier curve of aggregated estimated survival probability at different time points (12, 24 and 36 months) as well as median survival.

## Results

**Fig. 1:**
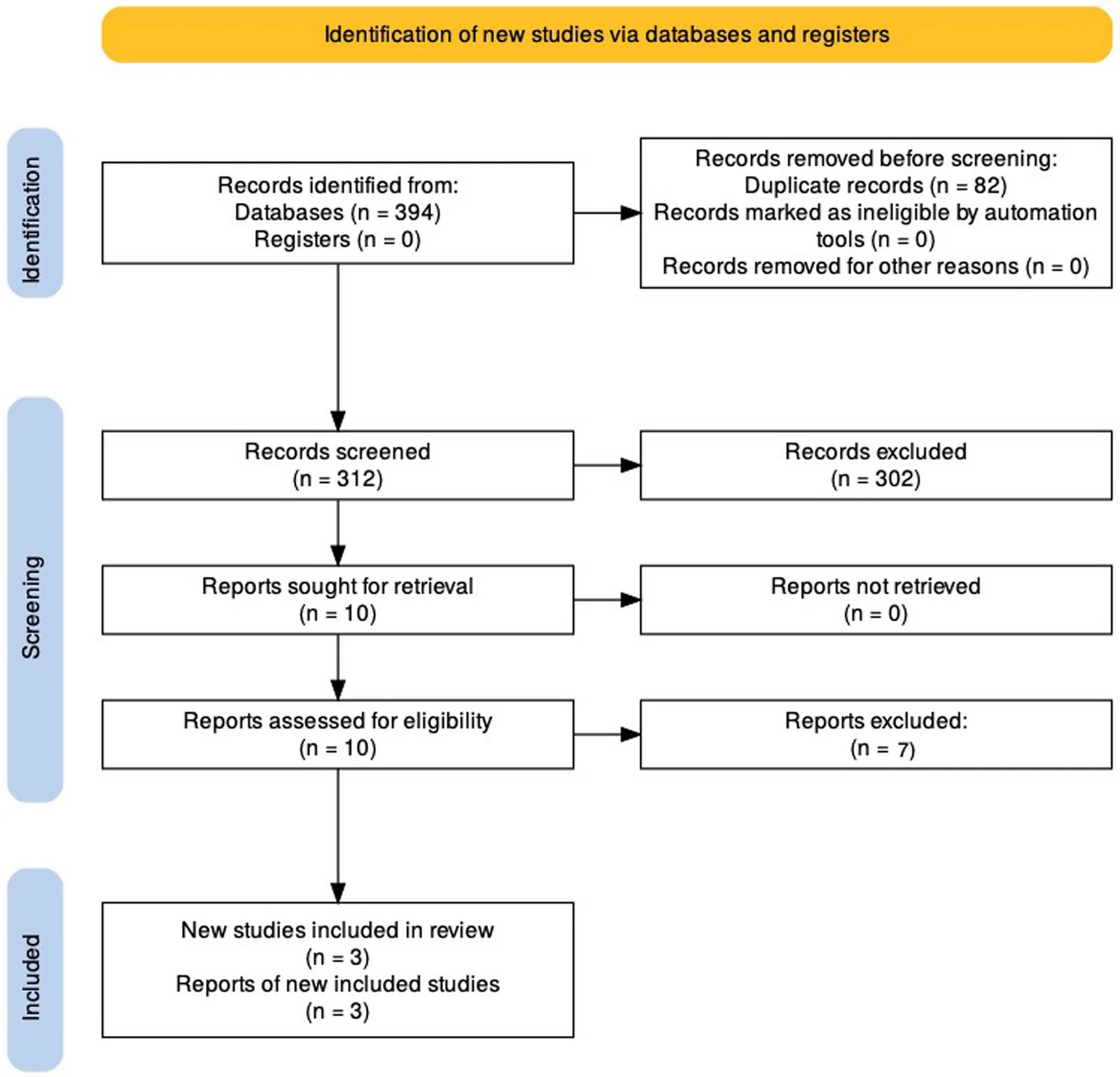
PRISMA flow diagram of study screening and selection. Blue vertical boxes indicate each stage of the screening process, and the horizontal boxes present detailed information on record identification, screening, eligibility assessment, and study inclusion. A total of 394 records were identified through database searches, with no records identified from registers. After removal of 82 duplicates, 312 records were screened, of which 302 were excluded. Ten reports were sought for retrieval and assessed for eligibility, resulting in three studies included in the systematic review and meta-analysis. Seven reports were excluded after full-text eligibility assessment

Our initial search yielded 394 results. After excluding duplicates and by titles and abstracts, 10 studies were fully assessed. Finally, we included three clinical trials (phase II or III) studies (Fig. 1).

Blue vertical boxes indicate each stage of the screening process, and the horizontal boxes present detailed information on record identification, screening, eligibility assessment, and study inclusion. A total of 394 records were identified through database searches, with no records identified from registers. After removal of 82 duplicates, 312 records were screened, of which 302 were excluded. Ten reports were sought for retrieval and assessed for eligibility, resulting in three studies included in the systematic review and meta-analysis. Seven reports were excluded after full-text eligibility assessment.

### Baseline Characteristics

Overall, 685 patients with advanced HER2-positive gastroesophageal adenocarcinoma were included in this systematic review and meta-analysis. The median age of patients ranged from 58 to 64 years. Patients were required to be previously untreated in advanced/metastatic setting. About 81.1% of patients were female (*n* = 556/685). The most common dosing regimen of Zanidatamab was 1800 mg in patients weighing < 70 kg and 2400 mg in those weighing ≥ 70 kg. The majority of the anatomical subtypes were gastric (67%; *n* = 459/685), followed by the gastroesophageal junction (22.7%; *n* = 156/685), and esophageal (10.2%; *n* = 70/685). A detailed description of individual study characteristics is presented in Table 1.

**Table 1:** Main characteristics of included studies

		HERIZON-GEA-01 (cohort B) (NCT05152147)	HERIZON-GEA-01 (cohort C) (NCT05152147)	Elimova et al. (NCT03929666)	Lee et al. (NCT04276493)
Location		Global		Canada, South Korea, USA	China, South Korea
Study design		Phase III, randomized, open-label		Phase II, single-arm, open-label	Phase Ib/II, single-arm, open-label
Enrolment period		December, 2021 to February, 2025		August, 2019 to February, 2022	March, 2020 to June, 2021
Median follow-up		26 months		47.9 months	30.1 months
Number of patients (n)		304	302	46	33
Regimen		Zanidatamab	Zanidatamab + Tislelizumab	Zanidatamab	Zanidatamab + Tislelizumab
Chemotherapy (n, %)	CAPOX	276 (90.8)	273 (90.4)	20 (43.4)	33 (100)
	FP	28 (9.2)	29 (9.6)	2 (4.3)	0 (0)
Median age		62.5	63.0	58	64
Male (n, %)		244 (80.2)	244 (80.7)	39 (84.7)	29 (87.8)
ECOG	0	134 (44.1)	121 (40.1)	26 (57)	11 (33.3)
	1	170 (55.9)	180 (59.6)	20 (43)	22 (66.7)
Disease status (n, %)	Unresectable locally advanced	9 (3.0)	18 (6.0)	8 (17.0)	0
	Advanced	295 (97.0)	284 (94.0)	38 (83.0)	33 (100)
Anatomical subtype (n, %)	Gastric	204 (67.1)	208 (68.9)	19 (41.0)	28 (84.8)
	GEJ	61 (20.1)	74 (24.5)	16 (35.0)	5 (15.2)
	Esophageal	39 (12.8)	20 (6.6)	11 (24.0)	0 (0)
PD-L1 status (n, %)	< 1%	108 (35.5)	90 (29.8)	NA	8 (24.2)
	> 1%	178 (58.6)	187 (61.9)	NA	24 (72.7)
HER2 status (n, %)	IHC 2+/ISH+	53 (17.4)	51 (16.9)	5 (11.0)	3 (9.1)
	IHC 3+	251 (82.6)	251 (83.1)	36 (78.0)	24 (72.7)

*CAPOX:* capecitabine + oxaliplatin regimen, *FP:* 5-fluorouracil (5-FU) and cisplatin (CDDP) regimen, *IHC:* immunohistochemistry, *ISH:* in situ hybridization, HER2 positivity was defined as IHC 3 + or IHC 2 + with ISH amplification (ISH +)

### Efficacy Endpoints

**Fig. 2:**
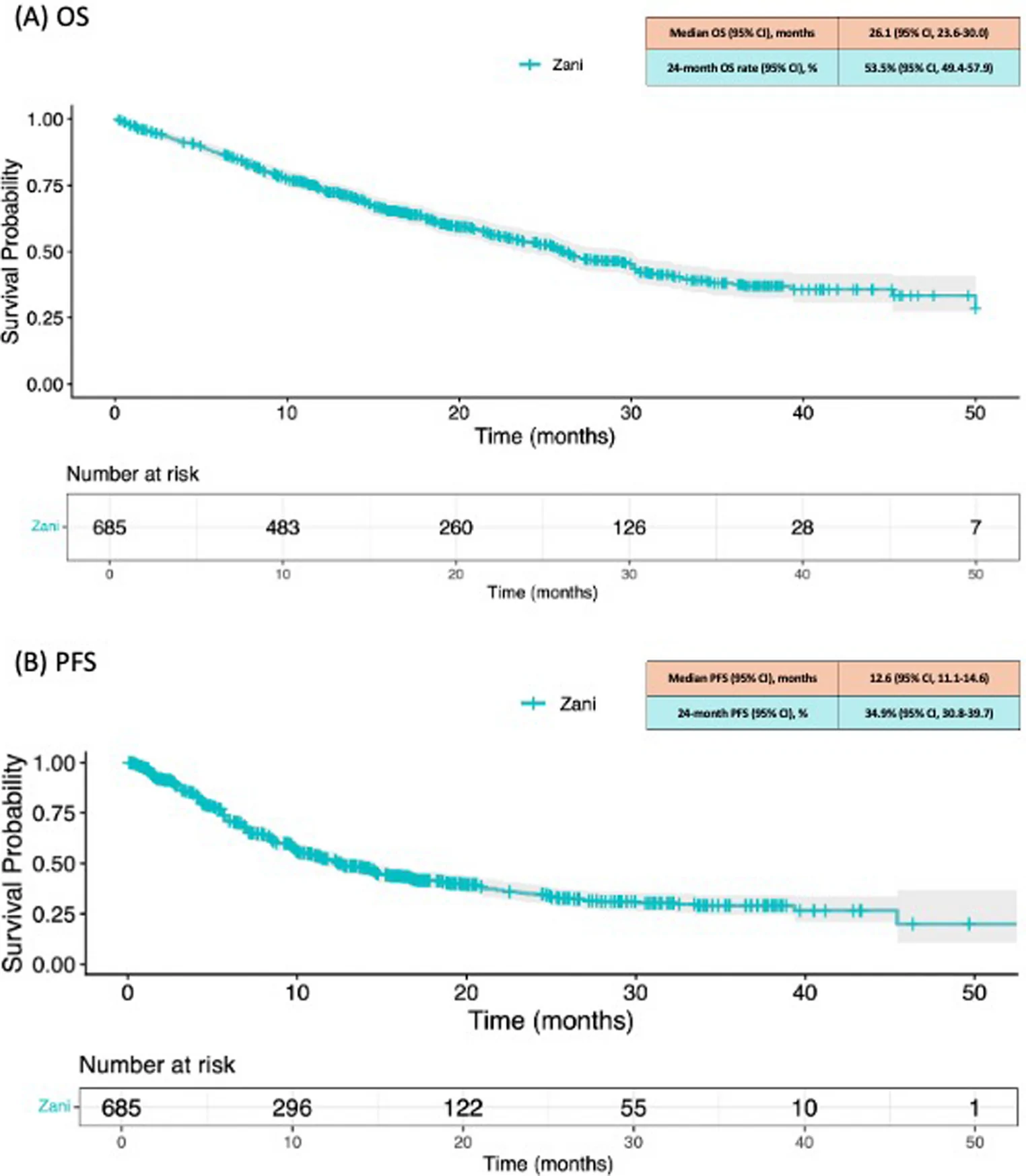
Reconstructed pooled survival outcomes of patients with advanced HER2-positive gastric or gastroesophageal junction adenocarcinoma treated with zanidatamab-based therapy. Kaplan–Meier curves represent reconstructed pooled estimates for overall survival and progression-free survival. Median OS was 26.1 months (95% CI, 23.6–30.0), with a 24-month OS rate of 53.5% (95% CI, 49.4–57.9). Median PFS was 12.6 months (95% CI, 11.1–14.6), with a 24-month PFS rate of 34.9% (95% CI, 30.8–39.7). CI: confidence interval; OS: overall survival; PFS: progression-free survival

**Fig. 3:**
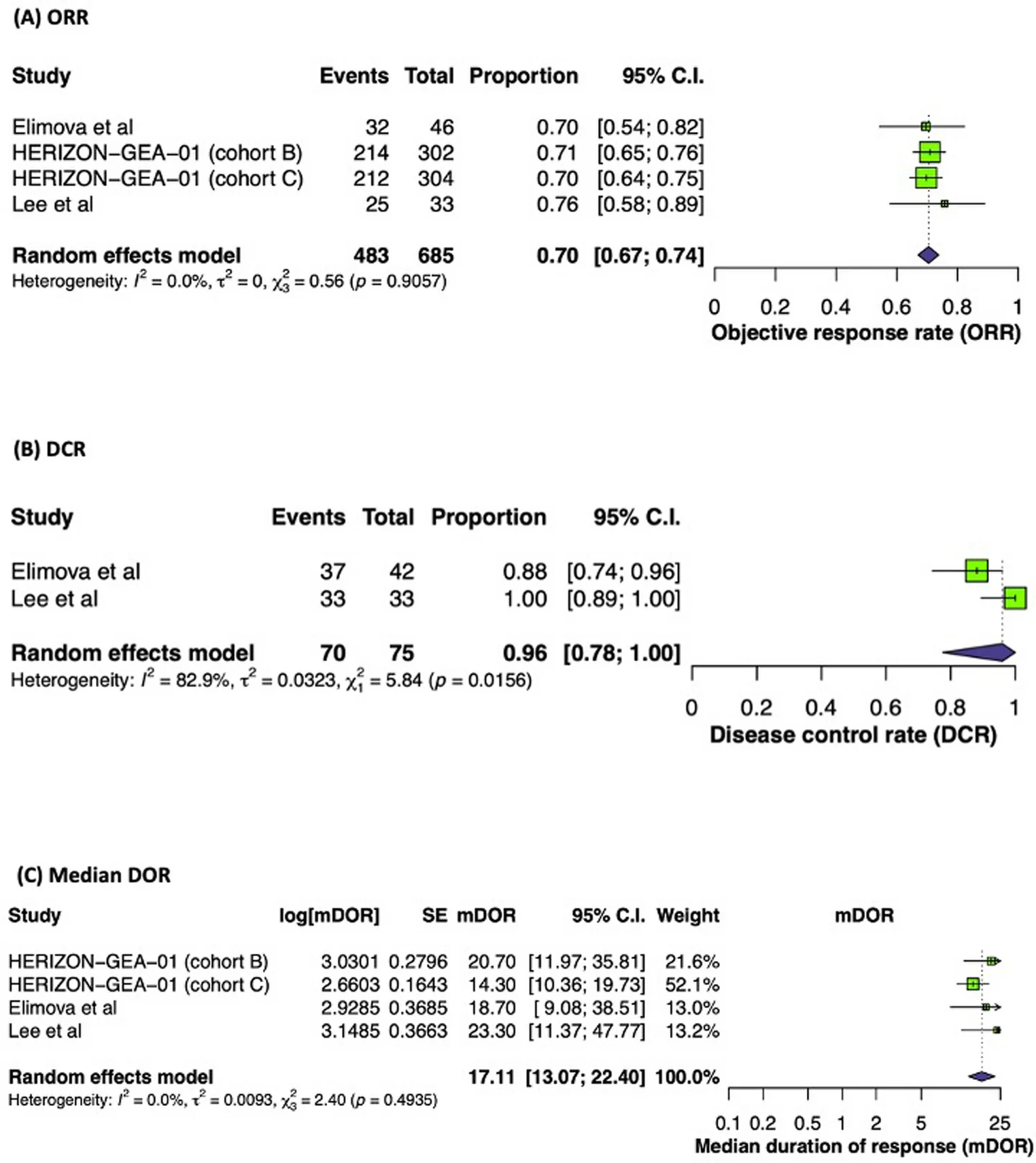
Pooled efficacy outcomes of zanidatamab-based therapy in advanced HER2-positive gastric or gastroesophageal junction adenocarcinoma. Pooled estimates are presented for objective response rate, disease control rate, and median duration of response. Squares represent study-level estimates, with size reflecting study weight, and horizontal lines indicate the 95% confidence intervals. Diamonds represent the estimated overall pooled effect of the meta-analysis. CI: confidence interval; DCR: disease control rate; DOR: duration of response; GEA: gastric or gastroesophageal junction adenocarcinoma; ORR: objective response rate

The reconstructed Kaplan-Meier survival curves and total number of events in each group are shown in Fig. 2. Overall survival data are described in Table 2. The reconstructed pooled median OS was 26.05 months (95% CI: 23.60-30.04), while the pooled median PFS was 12.61 months (95% CI: 11.05–14.60). OS rates at 12, 24, and 36 months were 73.5% (95% CI: 70.2%-77.0%), 53.5% (95% CI: 49.4%-57.9%), and 37.6% (95% CI: 32.9%-42.9%), respectively. Corresponding PFS rates were 51.9% (95% CI: 47.9%-56.1%), 34.9% (95% CI: 30.8%-39.7%), and 29.0% (95% CI: 24.6%-34.3%). Secondary efficacy outcomes included a pooled median ORR of 70% (*n* = 483/685; 95% CI: 67%-74%) and a DOR of 17.11 months (95% CI: 13.07–22.40 months), and a DCR of 96% (*n* = 70/75; 95% CI: 78%-100%) (Fig. 3).

**Table 2:** Survival data for patients under Zanidatamab

Outcome	Pooled median survival in months (95% CI)	Survival at 12 months, % (95% CI)	Survival at 24 months, % (95% CI)	Survival at 36 months, % (95% CI)
OS	26.1 (23.6–30.0)	73.5% (70.2–77.0%)	53.5% (49.4–57.9%)	37.6% (32.9–42.9%)
PFS	12.6 (11.1–14.6)	51.9% (47.9–56.1%)	34.9% (30.8–39.7%)	29.0% (24.6–34.3%)

*CI:* confidence interval, *PFS:* progression-free survival, *OS:* overall survival

### Safety Endpoints

**Fig. 4:**
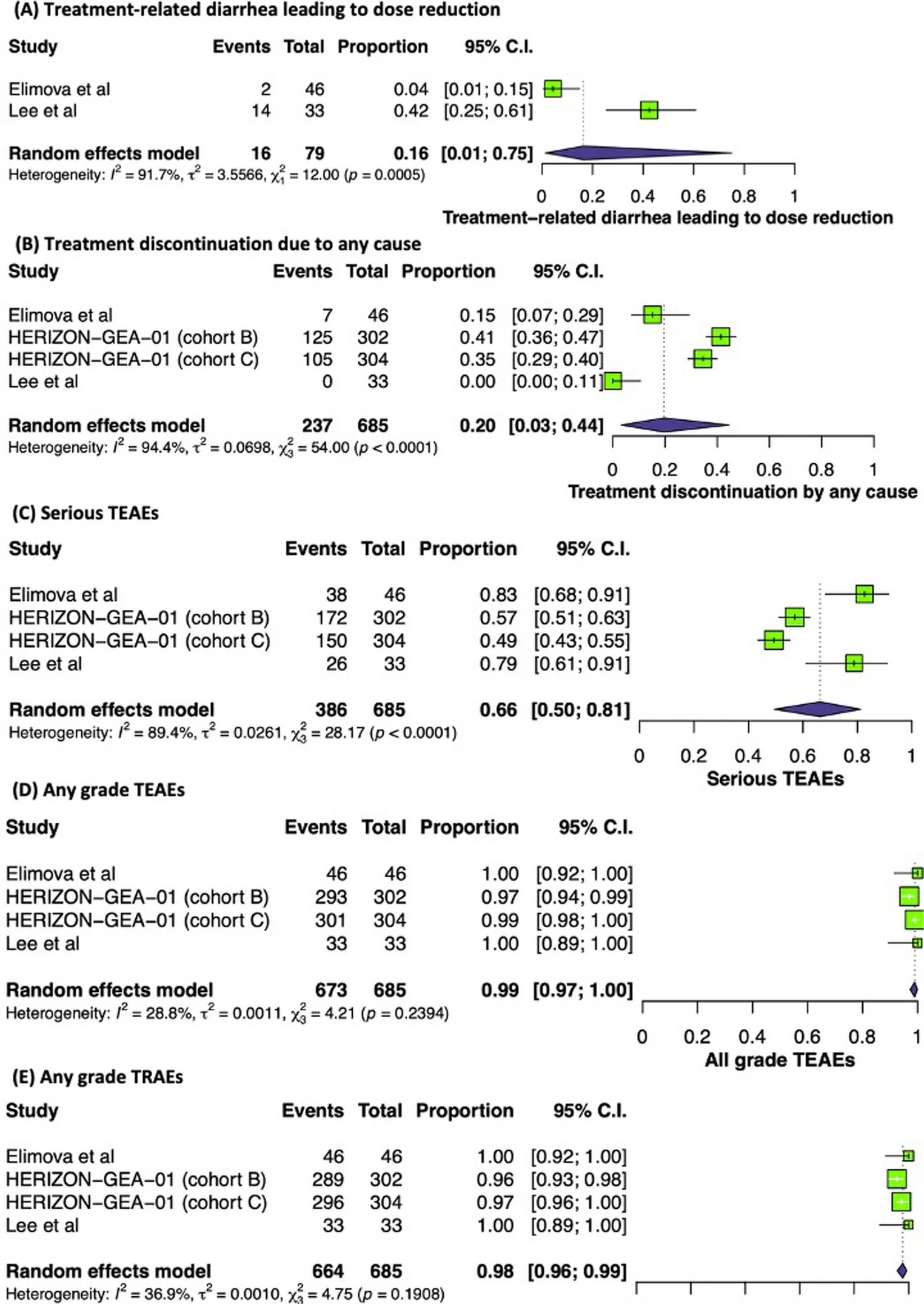
Pooled safety outcomes of zanidatamab-based therapy in advanced HER2-positive gastric or gastroesophageal junction adenocarcinoma. Pooled estimates are presented for treatment-related diarrhea leading to dose reduction, treatment discontinuation due to any cause, serious treatment-emergent adverse events, any-grade treatment-emergent adverse events, and any-grade treatment-related adverse events. Squares represent study-level estimates, with size reflecting study weight, and horizontal lines indicate the 95% confidence intervals. Diamonds represent the estimated overall pooled effect of the meta-analysis. CI: confidence interval; TEAEs: treatment-emergent adverse events; TRAEs: treatment-related adverse events

Treatment-related diarrhea leading to dose reduction occurred in 16% of patients (*n* = 16/79; 95% CI: 1%-75%). Treatment discontinuation due to any cause was observed in 20% of cases (*n* = 237/685; 95% CI: 3%-44%). Serious TEAEs were reported in 66% of patients (*n* = 386/685; 95% CI: 50%-81%). Overall, any-grade TEAEs and TRAEs occurred in 99% (*n* = 673/685; 95% CI: 97%-100%) and 98% (*n* = 664/685; 95% CI: 96%-99%) of patients, respectively (Fig. 4).

The incidence of common any-grade AEs was as follows: diarrhea (*n* = 549/685; 89%, 95% CI: 75%-98%), nausea (*n* = 360/685; 60%, 95% CI: 45%-74%), and infusion-related reactions (*n* = 179/685; 30%, 95% CI: 19%-43%) (Figure S1). Grade ≥ 3 toxicity was seen mainly with TRAEs (*n* = 443/685; 65%, 95% CI: 58%-71%) and diarrhea (*n* = 160/685; 26%, 95% CI: 19%-34%), whereas grade ≥ 3 nausea occurred in 5% (*n* = 4/79; 95% CI: 2%-13%) (Figure S2). Other clinically relevant AEs included immune-mediated AEs, with a pooled incidence of 25%, left ventricular dysfunction in 6%, and non-infectious pulmonary toxicity in 4% (Figure S3).

### Sensitivity Analysis

The leave-one-out analysis for ORR demonstrated results consistent with the overall effect, with pooled estimates ranging from 71% to 72% and no heterogeneity across analyses (I² = 0%), thereby supporting the robustness of the primary analysis.

### Quality Assessment

Among the two non-randomized studies, both lacked adjustment for confounding factors and failed to meet the specified criteria for the first and the sixth domain (Table S3A). Therefore, they were judged at a moderate risk of bias. HERIZON-GEA-01 was the only RCT included in this study, and it met most of the criteria for all domains that were determined to be at low risk of bias (Table S3B).

## Discussion

In this reconstructed IPD meta-analysis derived from published Kaplan–Meier curves, 685 patients with advanced HER2-positive GEA treated with zanidatamab in combination with chemotherapy and/or tislelizumab in the first-line setting were included. Across three clinical trials, we observed a pooled median OS of 26.05 months (95% CI, 23.60–30.04) and a pooled median PFS of 12.61 months (95% CI, 11.05–14.60).

The incorporation of biomarker-driven therapies has transformed the management of advanced GEA. HER2 overexpression or amplification occurs in approximately 15–20% of gastroesophageal adenocarcinomas and serves as a predictive biomarker for response to HER2-directed therapies. For more than a decade, trastuzumab combined with chemotherapy represented the standard first-line treatment following the ToGA trial [3]. More recently, KEYNOTE-811 established the addition of pembrolizumab to trastuzumab and chemotherapy as a new standard of care among patients with PD-L1 CPS ≥ 1 [6]. In the second-line setting, trastuzumab deruxtecan has become the preferred treatment option for patients with persistent HER2 positivity based on the phase III DESTINY-Gastric04 trial [10]. 

Zanidatamab represents a distinct approach to HER2 blockade. Unlike trastuzumab and pertuzumab, which each bind a single HER2 epitope, zanidatamab is a humanized bispecific biparatopic antibody that simultaneously targets the dimerization domain (ECD2) and the juxtamembrane domain (ECD4). This dual engagement promotes receptor clustering and internalization, resulting in enhanced inhibition of HER2 signaling and increased immune-mediated antitumor activity. Preclinical studies have demonstrated a unique pattern of HER2 reorganization on the cell surface that is not observed with trastuzumab, pertuzumab, or their combination [1, 7]. 

Consistent with these biological properties, zanidatamab-based therapy demonstrated substantial antitumor activity in our analysis, with a pooled ORR of 70% and a median DOR of 17.1 months. Interpretation according to HER2 expression level was limited by the absence of subgroup data for patients with IHC 2+/ISH-positive tumors, despite this subgroup representing approximately 16% of the pooled study population. Notably, HERIZON-GEA-01 reported generally consistent overall survival outcomes across PD-L1-defined subgroups, including patients with PD-L1-negative tumors [1]. Whether this observation is related to the enhanced immune-mediated effects associated with dual HER2 targeting remains uncertain and was not evaluated in the present analysis.

Despite promising efficacy, zanidatamab-based treatment had a toxicity burden, with grade ≥ 3 adverse events in 65% of patients. Diarrhea was the main cause of dose reductions, with prophylaxis mandated in III trial. Treatment discontinuation due to zanidatamab was infrequent. Adverse events included immune mediated toxicities, left ventricular dysfunction, and non-infectious pulmonary toxicity, some possibly related to concomitant immune checkpoint inhibition.

This study has several limitations. First, although 685 patients were included, the pooled estimates were largely driven by HERIZON-GEA-01, the only phase III trial, whereas the remaining studies were smaller early-phase investigations. Second, treatment regimens were not uniform across studies, with some patients receiving zanidatamab in combination with chemotherapy alone and others receiving zanidatamab, chemotherapy, and tislelizumab, limiting the ability to isolate the contribution of zanidatamab. Third, this analysis was based on reconstructed individual patient data derived from published Kaplan–Meier curves rather than original patient-level datasets, which may introduce inaccuracies. Moreover, the pooled analysis of median DOR was carried out using log-transformed values from median estimates as Kaplan-Meier curves for this outcome were unavailable for reconstruction. Although, our sensitivity using the log-transformed values from median PFS yielded a consistent effect size compared to the one seen in the reconstructed IPD analysis, we acknowledge that log-transformed estimates may be affected by different follow-up times and censoring across studies. In addition, the small number of available studies precluded robust assessment of publication bias and limited subgroup analyses according to clinically relevant factors such as HER2 expression level and PD-L1 status. Furthermore, several efficacy and safety outcomes were reported in only a subset of studies, resulting in wider confidence intervals for some pooled estimates. Lastly, our analysis was restricted to data derived from clinical trials, and no real-world studies evaluating zanidatamab in this setting were identified in our search.

The therapeutic landscape of HER2-positive GEA continues to evolve rapidly. Trastuzumab deruxtecan is currently being evaluated in earlier treatment settings, including in combination with chemotherapy and pembrolizumab in DESTINY-Gastric03, where dose optimization strategies have sought to improve tolerability while maintaining efficacy [11]. Additional HER2-directed approaches under investigation include novel monoclonal antibodies such as HLX22 (NCT04908813), antibody-drug conjugates such as disitamab vedotin (NCT05980481), bispecific immune checkpoint inhibitors such as rilvegostomig (NCT06764875), and vaccine-based approaches such as HER-Vaxx (NCT02795988).

Another area of growing interest is the potential role of HER2-directed therapies in HER2-low GEA. While HER2-low disease has emerged as a clinically relevant entity in breast cancer, leading to the incorporation of ADCs into routine practice, the evidence in gastric cancer remains considerably less mature [12]. The applicability of the HER2-low paradigm to GEA is complicated by the marked spatial and temporal heterogeneity of HER2 expression. Nevertheless, early studies with ADCs, particularly trastuzumab deruxtecan and disitamab vedotin, have demonstrated signals of activity in tumors with low levels of HER2 expression, supporting further investigation of this population [13, 14]. 

## Conclusion

This reconstructed IPD meta-analysis derived from published Kaplan–Meier curves indicate that first-line therapy involving zanidatamab shows significant antitumor effects in advanced HER2-positive GEA, with promising results in overall survival, progression-free survival, response rate, and response duration. Nonetheless, its promising efficacy must be considered alongside a notable toxicity profile, especially grade ≥ 3 treatment-related adverse events. More mature data and additional randomized studies are needed to better understand zanidatamab’s specific efficacy, its role in biomarker-defined subgroups, and to better select patients in this rapidly evolving landscape.

## Supplementary Information

Below is the link to the electronic supplementary material.

Supplementary Material 1

## Data Availability

No datasets were generated or analysed during the current study.
